# 1′-Acetoxychavicol Acetate Isolated from *Alpinia galanga* Ameliorates Ovalbumin-Induced Asthma in Mice

**DOI:** 10.1371/journal.pone.0056447

**Published:** 2013-02-25

**Authors:** Joung-Wook Seo, Soon-Chang Cho, Sang-Joon Park, Eun-Ji Lee, Jong-Hwa Lee, Sang-Seop Han, Byeong Sik Pyo, Dae-Hun Park, Bong-Hee Kim

**Affiliations:** 1 Korea Institute of Toxicology, Daejeon, Korea; 2 College of Pharmacy, Chungnam National University, Daejeon, Korea; 3 Center for Senior Industry, Youngdong University, Yeongdong, Korea; 4 College of Veterinary Medicine, Kyungpook National University, Daegu, Korea; 5 College of Veterinary Medicine, Chungnam National University, Daejeon, Korea; 6 Konyang University, Daejeon, Korea; 7 Dongshin University, Naju, Korea; University of Rochester Medical Center, United States of America

## Abstract

The World Health Organization reports that 235 million people are currently affected by asthma. This disease is associated with an imbalance of Th1 and Th2 cells, which results in the upregulation of cytokines that promote chronic inflammation of the respiratory system. The inflammatory response causes airway obstruction and can ultimately result in death. In this study we evaluated the effect of 1′-acetoxychavicol acetate (ACA) isolated from *Alpinia galanga* rhizomes in a mouse model of ovalbumin (OVA)-induced asthma. To generate the mouse model, BALB/c mice were sensitized by intraperitoneal injection of OVA and then challenged with OVA inhalation for 5 days. Mice in the vehicle control group were sensitized with OVA but not challenged with OVA. Treatment groups received dexamethasone, 25 mg/kg/day ACA, or 50 mg/kg/day ACA for 5 days. Asthma-related inflammation was assessed by bronchoalveolar lavage fluid cell counts and histopathological and immunohistochemical analysis of lung tissues. Our results showed that ACA reduced the infiltration of white blood cells (especially eosinophils) and the level of IgE in the lungs of mice challenged with OVA and suppressed histopathological changes such as airway remodeling, goblet-cell hyperplasia, eosinophil infiltration, and glycoprotein secretion. In addition, ACA inhibited expression of the Th2 cytokines interleukin (IL)-4 and IL-13, and Th1 cytokines IL-12α and interferon-γ. Because asthmatic reactions are mediated by diverse immune and inflammatory pathways, ACA shows promise as an antiasthmatic drug candidate.

## Introduction

The World Health Organization reports that 235 million people are affected by asthma, which is the most common chronic disease among children. Triggers for asthma include indoor allergens (e.g., pet dander and dust mites in bedding, carpets, and stuffed furniture), outdoor allergens (e.g., pollens and molds), tobacco smoke, chemical irritants, and air pollution. Asthma is a serious disease that can result in death if not treated properly [1]. This chronic inflammatory lung disease causes bronchoconstriction, bronchial mucosal thickening from edema, eosinophilic infiltration, bronchial wall remodeling, and excessive mucus production, and can ultimately lead to airway obstruction [2], [3].

Asthma is an immune-mediated disease in which T helper (Th) cells play an important role. Mouse Th clones can be divided into two subsets according to cytokine secretion patterns [4]: Th1 cells secret interleukin (IL)-2 and interferon (IFN)-γ, and Th2 cells secrete IL-4, IL-13, and IL-5. In addition, Th2 cells promote B cell differentiation and class switching from Ig G to Ig E [5]. The cytokine IL-6 regulates the functions of CD4 T cells and mediates asthma induction [6], whereas IL-12 regulates the Th1/Th2 balance [7] and promotes IFN-γ production [8]. IFN-γ is related to the persistence and severity of asthma [9]. IL-4 and IL-13, which are key cytokines in the pathogenesis of asthma [10], are involved in airway remodeling, inflammatory processes, airway hyperresponsiveness, goblet-cell hyperplasia, eosinophil infiltration, mucus hypersecretion, and B cell activation [11], [12], [13], [14]. IL-5 regulates the development, activation, migration, and survival of eosinophils, which are characteristic features of asthma [15].

Asthma is controlled with bronchodilators, corticosteroids, leukotriene modifiers, theophylline, and/or anti-IgE therapy; however, none of these treatments are curative [16]. Inhaled corticosteroids are commonly used [17], but in addition to their side effects, these drugs tend to reduce glucocorticoid receptor-binding affinity and T-cell response [18]. Therefore, alternative therapies are sought from traditional medicines or other natural products that have therapeutic effects in respiratory disorders.

*Alpinia galangal* is a member of the ginger family but differs from *Zingiber officinale*, which is commonly used in Western cuisine. *Alpinia galanga* rhizomes have been traditionally used to treat bronchial problems in tropical areas of south and east India. This rhizome has also been reported to be useful as a carminative and as a treatment for rheumatoid arthritis, inflammation, stomatopathy, pharyngopathy, cough, asthma, hiccough, dyspepsia, stomachalgia, obesity, diabetes, cephalalgia, tubercular glands, and intermittent fevers [19]. Biological properties of *A. galanga* and its constituents include anticarcinogenic effects [20], chemoprevention through COX-2 suppression [21], antioxidative actions [22], and inhibition of TNF-α and IL-4 [23]. In addition, hydroxychavicol acetate, which is one of the constituents of *A. galanga*, increases IL-2 production and attenuates IFN-γ expression [24].

Although studies have reported that *A. galanga* exerts various biological effects and modulates inflammation, no studies have evaluated the ability of *A. galanga* to cure or completely control asthma. Therefore, in this study we evaluated the effect of 1′-acetoxychavicol acetate (ACA; 25 or 50 mg/kg/day) isolated from *A. galanga* on asthma using a mouse model of ovalbumin (OVA)-induced asthma.

## Results

### ACA reduced the number of eosinophils and other white blood cells and the level of IgE in bronchoalveolar lavage fluid

**Figure 1:**
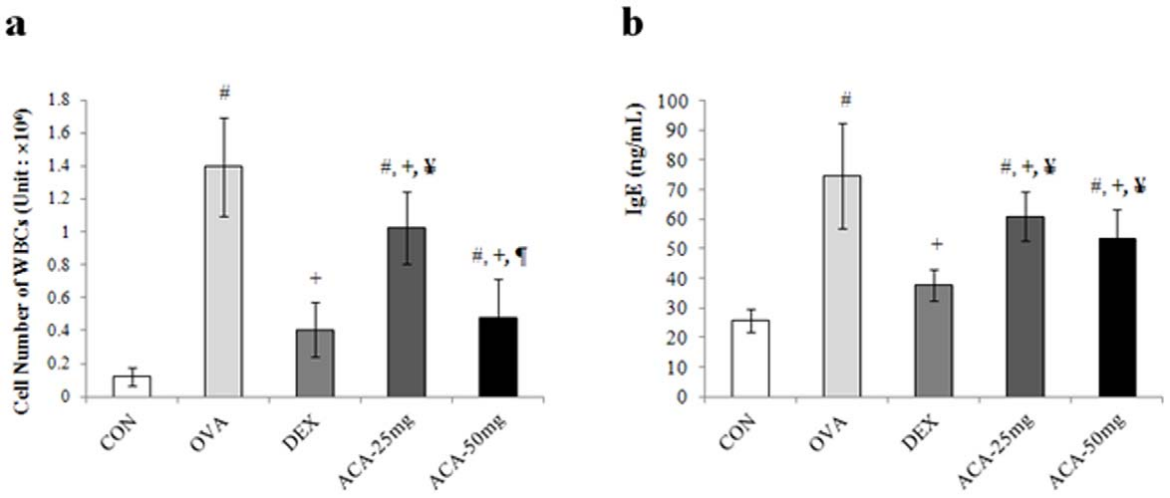
ACA dose-dependently decreased white blood cell counts and the level of IgE in the bronchoalveolar lavage fluid of mice with OVA-induced asthma. (a) ACA dose-dependently decreased white blood cell counts in the bronchoalveolar lavage fluid of mice with OVA-induced asthma. (b) ACA decreased IgE levels in the bronchioalveolar lavage fluid of mice with OVA-induced asthma. #*p*<0.01 vs. CON (vehicle control); +*p*<0.01 vs. OVA (OVA-induced asthma model); ¥*p*<0.01 vs. DEX (dexamethasone); ¶*p*<0.01 vs. ACA-25 mg (25 mg/kg/day ACA).

In the mouse model of OVA-induced asthma, the number of white blood cells (WBCs) in bronchoalveolar lavage fluid (BALF) was significantly increased compared with that of mice treated with vehicle alone (vehicle control) (Figure 1a). In addition, ACA dose-dependently decreased the number of WBCs, and mice treated with 50 mg/kg/day ACA had WBC counts similar to those of the vehicle-treated and dexamethasone-treated controls. Similarly to the result of WBCs counting in BALF, the level of IgE in ACA treated groups decreased although that of dexamethasone-treated controls was not suppressed (Figure 1b).

Asthma is characterized by eosinophilia. Accordingly, the number of eosinophils in BALF was significantly increased in the OVA-induced asthma model (Table 1). Our results showed that both doses of ACA (25 and 50 mg/kg/day) suppressed eosinophil infiltration. In particular, the number of eosinophils in the BALF of mice treated with 50 mg/kg/day ACA was similar to that of dexamethasone-treated mice. Lymphocyte levels were also elevated in the BALF of mice with OVA-induced asthma. Although the lower dose of ACA did not reduce this effect, the number of lymphocytes recovered in the BALF of mice treated with 50 mg/kg/day ACA was similar to that of mice in the vehicle control group.

**Table 1:** ACA reduced eosinophil numbers in bronchoalveolar lavage fluid recovered from mice with OVA-induced asthma.

	NEUs	LYMs	EOSs	BASs	LUCs
	(×10^6^)	(×10^6^)	(×10^6^)	(×10^6^)	(×10^6^)
Vehicle control	0.03±0.009	0.03±0.011	0.01±0.012	0.01±0.004	0.05±0.040
Albumin-induced asthma model	0.04±0.012	0.11±0.052 a	1.14±0.270 a	0.01±0.004	0.10±0.023
Dexamethasone treatment	0.02±0.005	0.03±0.010 ^b^	0.27±0.150 ^b^	0.01±0.002	0.08±0.034
25 mg/kg/day ACA treatment	0.07±0.077	0.08±0.049	0.73±0.299 a,b,c	0.01±0.023	0.13±0.059 a
50 mg/kg/day ACA treatment	0.02±0.007	0.04±0.019 ^b^	0.34±0.198 ^b,d^	0.01±0.004	0.07±0.019

Results are expressed as mean ± SD (n = 7). NEU, neutrophils; LYMs, lymphocytes; EOSs, eosinophils; BASs, basophils; LUCs, large unstained cells.

a *p*<0.01 vs. CON (vehicle control); ^b^ *p*<0.01 vs. OVA (OVA-induced asthma model); ^c^ *p*<0.01 vs. DEX (dexamethasone treatment); ^d^ *p*<0.01 vs. ACA-25 mg (25 mg/kg/day ACA treatment).

### ACA dose-dependently inhibited OVA-induced histopathological changes in lung tissue

**Figure 2:**
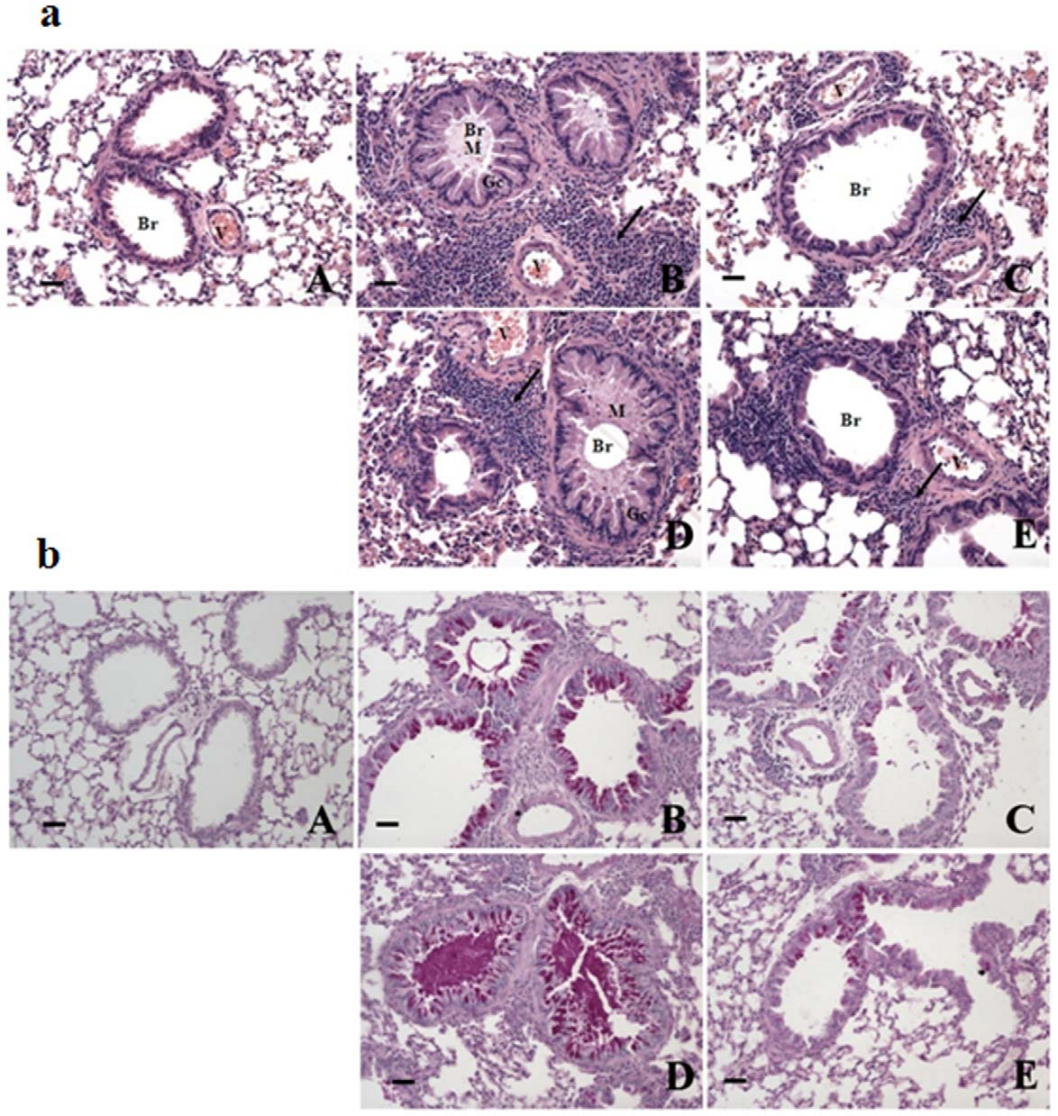
ACA dose-dependently inhibited histopathological changes in the lungs of mice with OVA-induced asthma. (a) ACA dose-dependently reduced inflammatory cell infiltration around vessels and bronchioles, mucus secretion and cell debris in bronchioles, and goblet cell hyperplasia in the lungs. Bar size, 50 μm; hematoxylin and eosin stain. (b) ACA dose-dependently decreased bronchial secretion of glycoproteins in OVA-induced asthma. Bar size, 50 μm; PAS stain. Arrow: inflammatory cell infiltration. Br, bronchiole; Gc, goblet cell; M, mucus secretion; V, vessel. A, vehicle control; B, OVA-induced asthma model; C, dexamethasone; D, 25 mg/kg/day ACA; E, 50 mg/kg/day ACA.

We observed histopathological changes in the lungs of mice with OVA-induced asthma compared with the vehicle control group (Figure 2a). These changes, which included airway remodeling, goblet-cell hyperplasia, eosinophil infiltration, and mucus plugs, were not completely prevented by 25 mg/kg/day ACA. However, the lung tissue of mice treated with 50 mg/kg/day ACA was similar to that of the vehicle control and dexamethasone treatment groups. Thus OVA-induced pathological changes in the lungs were suppressed by ACA in a dose-dependent manner.

Glycoprotein secretion was assessed by using the periodic acid-Schiff (PAS) stain (Figure 2b) and hematoxylin and eosin stain. Our results show that 25 mg/kg/day ACA partially suppressed OVA-induced glycoprotein secretion, and 50 mg/kg/day ACA reduced the level of glycoprotein secretion to that of the vehicle control group. Thus ACA dose-dependently suppressed glycoprotein secretion in mice with OVA-induced asthma.

### ACA suppressed T cells but had little or no effect on B cells

**Figure 3:**
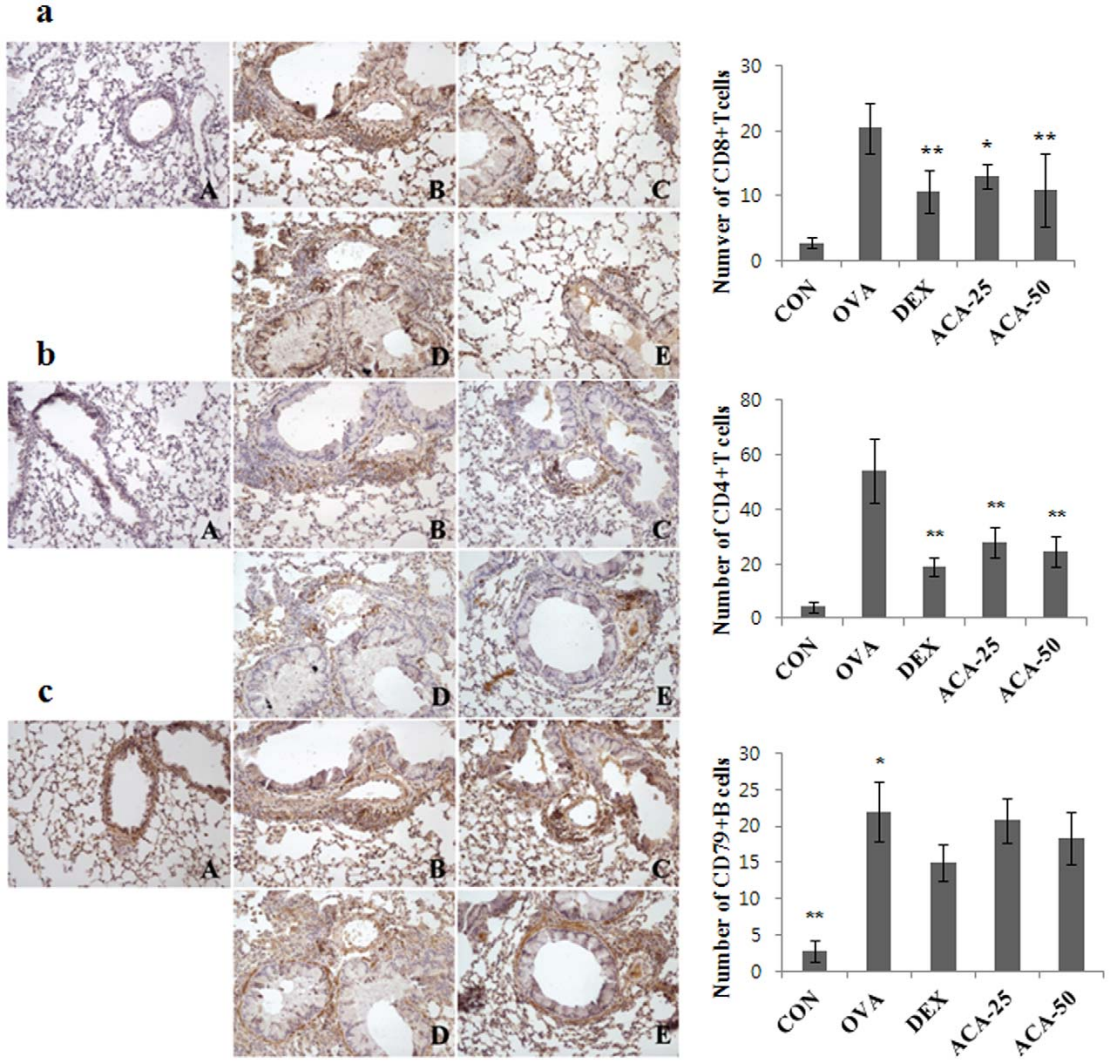
ACA suppressed T cells but not B cells in mice with OVA-induced asthma. (a) ACA dose-dependently suppressed the upregulation of CD8+ cytotoxic T cells in the lungs. (b) ACA suppressed the upregulation of CD4+ Th cells as effectively as dexamethasone. (c) ACA did not affect CD79α+ B cells in the lungs of mice with OVA-induced asthma. Immunopositive cells were counted in five randomly selected nonoverlapping fields (×200 magnification) of three separately immunostained lung sections per animal. A, vehicle control; B, OVA-induced asthma model; C, dexamethasone; D, 25 mg/kg/day ACA; E 50 mg/kg/day ACA.

Allergen-induced asthma consists of early and late responses mediated by immune cells (e.g., Th cells and B cells) and the cytokine cascade [25]. Therefore, we characterized infiltrating lymphocytes by immunohistochemistry using specific T and B cell markers such as CD4, CD8 and CD79. Increased secretion of IL-4 and IL-13 by T cells leads to antibody class switching (from IgG to IgE) by B cells, and IL-5 induces eosinophilia [26]. As shown in Figure 3a, the OVA-induced increase in CD8+ cytotoxic T cells was dose-dependently suppressed near bronchial and pulmonary arteries by ACA treatment. Infiltration of CD4+ Th cells, which are important in the pathogenesis of asthma, was much more increased than that of CD8+ T cells in the OVA-induced asthma model, and this response was also suppressed by ACA (Figure 3b). In contrast, expression of CD79α, a marker of B cell activation, was not altered by ACA (Figure 3c). The results show that ACA is more effective at suppressing CD8 cytotoxic T cells and CD4 Th cells than B cells.

### ACA suppressed expression of cytokines related to Th1/2 cells in OVA-induced asthma

**Figure 4:**
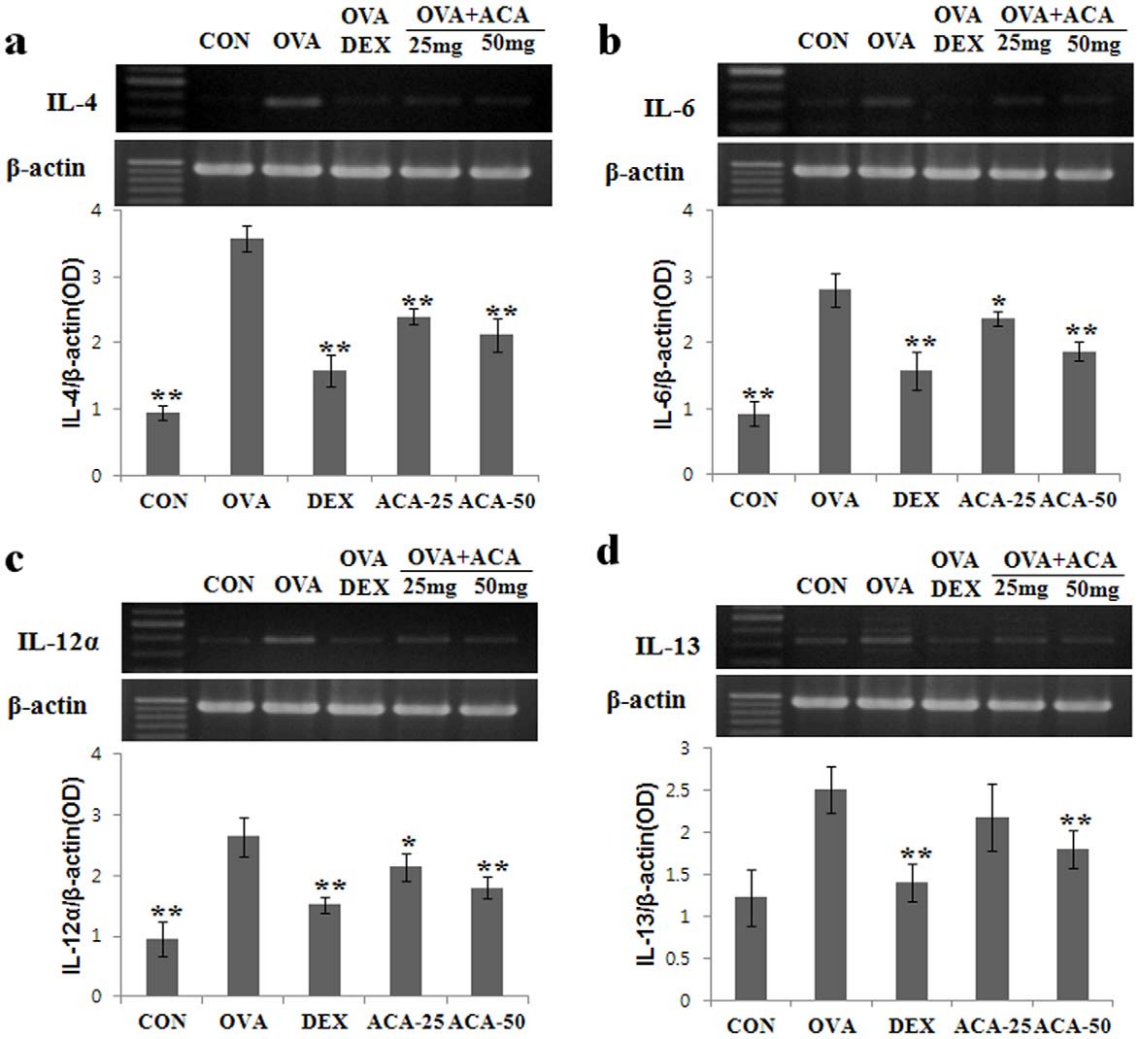
ACA dose-dependently suppressed expression of several Th1/2 cytokines in OVA-induced asthma. (a) IL-4 mRNA levels. (b) IL-6 mRNA levels. (c) IL-12α mRNA levels. (d) IL-13 mRNA levels. Results are expressed as mean ± SD (n = 6 per group). *p<0.05 and **p<0.01 vs. OVA group.

During allergic asthmatic inflammation and airway remodeling, recruited inflammatory cells, lung epithelial cells, and resident lung macrophages are activated and release cytokines promoting a Th2 type immune response in the lungs. To investigate this effect, we examined whether ACA treatment could alter the expression of Th1/2 cytokines IL-4, IL-6, IL-12α, and IL-13 in lung tissues. We focused on the expression of Th1/2 cytokines because immunohistochemistry results showed that T cells were reduced after ACA treatment. In the present study, expression of Th2 cytokines IL-4, IL-6, and IL-13 were decreased dose-dependently in the ACA-treated mice compared with the untreated OVA-challenged group (Figure 4a, 4b, and 4d). In addition, the Th1 cytokine IL-12α was decreased in ACA-treated mice compared with the untreated OVA-challenged group (Figure 4c). Thus, ACA treatment influences the cytokine milieu in the allergic asthmatic state.

### ACA reduced expression of Th2 and Th1 cytokines

**Figure 5:**
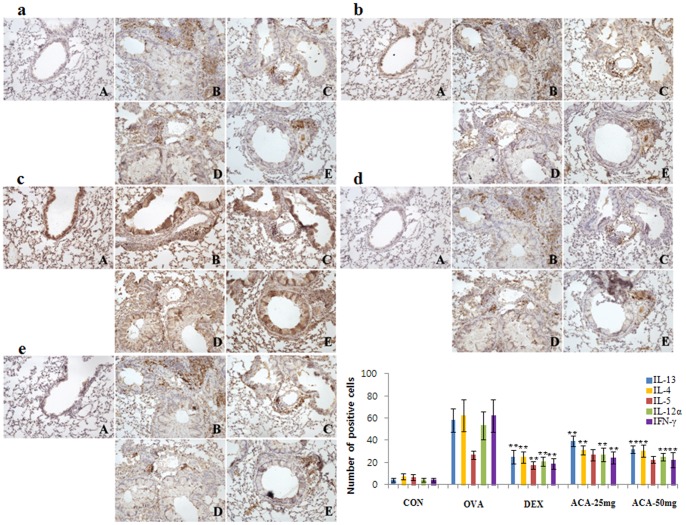
ACA reduced expression of Th2 and Th1 cytokines in OVA-induced asthma. Treatment with ACA reduced (a) IL-13 expression and (b) almost completely blocked IL-4 expression in the lungs. (c) ACA also decreased IL-5 expression but to a lesser extent. (d) ACA almost completely blocked IL-12α expression and (e) downregulated IFN-γ expression. Immunopositive cells were counted in five randomly selected nonoverlapping fields (×200 magnification) of three separately immunostained lung sections per each animal. A, vehicle control; B, OVA-induced asthma model; C, dexamethasone; D, 25 mg/kg/day ACA; E, 50 mg/kg/day ACA. Results are expressed as mean ± SD (n = 7 per group);*p<0.05 and **p<0.01 vs. OVA group.

Asthma is characterized by increased secretion of proinflammatory cytokines by Th2 and Th1 cells [9]. We further investigated the localization and number of infiltrated inflammatory cells responsible for cytokine expression. Cytokines localized primarily near inflamed bronchial and pulmonary arterioles. Th2 cytokines IL-13 and IL-4, which were overexpressed in the OVA-induced asthma model, were suppressed by both doses of ACA (Figure 5a and 5b). However, ACA did not significantly inhibit OVA-induced overexpression of IL-5 (Figure 5c.) In addition, ACA suppressed the secretion of Th1 cytokines IL-12α and IFN-γ (Figure 5d and 5e).

## Discussion

In our study, we found that ACA dose-dependently suppressed WBC infiltration of the lungs in mice with OVA-induced asthma, and 50 mg/kg/day ACA treatment reduced the WBC count to that of the vehicle control group. Specifically, eosinophil infiltration, which is characteristic of asthma, was significantly suppressed by ACA. In addition, ACA blocked OVA-induced histopathological changes such as airway remodeling, goblet-cell hyperplasia, eosinophil infiltration, and mucus plugs. Although treatment with ACA did not inhibit B cell activation, as assessed by CD79α expression, our results show that ACA is effective at reducing populations of CD4+ Th cells and CD8+ cytotoxic T cells in the lungs of mice with OVA-induced asthma. Finally, ACA downregulated Th2 cytokines IL-4 and IL-13 and Th1 cytokines IL-12α and IFN-γ, but did not affect the secretion of IL-5.

The relationship between Th1 cells and Th2 cells plays an important role in the pathogenesis of asthma. Mamessier and Magnan [9] hypothesized that there are three situations related to asthma. In a healthy subject, activation of Th1 and Th2 cells is balanced, and the level of regulatory T-cell activation is relatively low. In well-controlled asthma, the level of Th1 cell activation is similar to that of regulatory T cells, but Th2 cell activation is suppressed. In uncontrolled asthma, the level of Th2 cell activation is lower than that of Th1 cells, which in turn is lower than that of regulatory T cells. Thus, not only is the balance between Th1 and Th2 cells important, equilibrium is needed between Th1/ Th2 cells and regulatory T cells.

The Th2 cytokines IL-4 and IL-13 promote acute inflammatory processes in the pathogenesis of asthma and structural changes in the airways; [10], [11], [27]. We found that ACA dose-dependently reduced IL-4 and IL-13 levels in the lungs (Figure 5d). In addition, ACA decreased IL-12 α and INF-γ levels as effectively as dexamethasone (Figure 5e). Asthma was traditionally though to be initiated by an imbalance between Th1 and Th2 cells, The functions of IL-12 have been fairly well characterized; however, the role of INF-γ in asthma has been controversial. Although *Caenorhabditis elegans* extract was reported to ameliorate asthma symptoms by increasing INF-γ expression, hydrocortisone, which is used to treat asthma, has been shown to decrease INF-γ expression [28]. Previous studies have reported elevated INF-γ levels in the BALF and bronchioles of asthma patients [29], [30]. In addition, airway hyperresponsiveness after methacholine challenge was more severe in IFN-γ transgenic mice than in normal mice [31]. Our finding that ACA decreased INF-γ expression in OVA-induced asthma suggests that ACA suppresses Th1-related cytokines as well as Th2 cytokines.

Although steroids cause a variety of adverse effects, they can inhibit proinflammatory responses and induce anti-inflammatory gene expression. Asthma therapies that target multiple pathways are more likely to be effective than therapies that modulate a single target, because asthmatic reactions are mediated by numerous immune and inflammatory pathways. Because ACA inhibits various proinflammatory cytokines, it shows promise as an antiasthmatic drug candidate.

## Materials and Methods

### Plant material

Dried *A. galanga* rhizomes were purchased from Dermalab, Gyeonggi, Korea in May 2010. A voucher sample was deposited at the Center for Senior Industry of the Youngdong University (identification number: YD1202).

### Isolation of ACA

**Figure 6:**
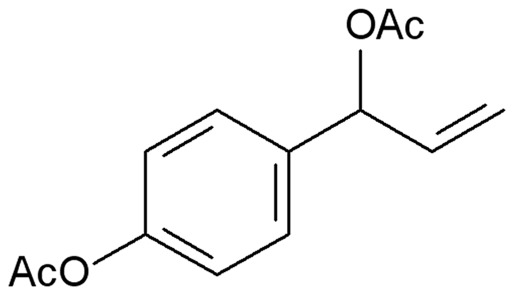
Chemical structure of 1′-acetoxychavicol acetate.

The dried *A. galanga* rhizomes (3 kg) were chopped and then extracted twice with 90% aqueous methanol (15 L) in a shaker (90 rpm) at 30°C for 2 days. The methanol extracts were combined and concentrated under vacuum, suspended in water (700 mL), and then extracted four times with 300 mL ethyl acetate. The ethyl acetate layer was concentrated, yielding a brown oily substance (69 g), which was chromatographed on a silica gel column (230–400 mesh, 150 id×400 mm) by stepwise elution with methylene chloride and methanol mixtures of increasing polarity, yielding 12 fractions by thin layer chromatography monitoring. Fraction 2 (22 g) was further purified by high-performance liquid chromatography (YMC-Pack Pro C-18 column, S-5 μm, 20 id×250 mm; 40%–70% aqueous acetonitrile for 90 min, 7 mL/min) to yield 19.5 g ACA with >98% purity. ACA (Figure 6) is a colorless oil: ^1^H nuclear magnetic resonance (CD3OD, 400 MHz): δ 7.37 (2H, d, J = 8.6 Hz), 7.09 (2H, d, J = 8.6 Hz), 6.23 (1H, d, J = 6.0 Hz), 6.02 (1H, ddd, J = 17.2, 10.4, 6.0 Hz), 5.28 (1H, dd, J = 17.2, 1.2 Hz), 5.23 (1H, d, J = 10.4, 1.2 Hz), 2.26 (3H, s), 2.08 (3H, s); ^13^C nuclear magnetic resonance (CD3OD, 400 MHz): δ 170.2, 169.6, 150.6, 136.7, 136.2, 127.9, 121.5, 115.9, 75.7, 19.6, 19.5.

### Animal experiments

We conducted two animal studies using the same methods. Seventy female BALB/c mice were purchased from Orient Bio Inc. (Seungnam, Korea) and divided into five groups according to treatment: (1) 0.5% methylcellulose (Sigma Chemical Co., St. Louis, MO) as a vehicle control, (2) OVA-induced asthma model, (3) 1 mg/kg/day dexamethasone, (4) 25 mg/kg/day ACA, and (5) 50 mg/kg/day ACA. On days 1 and 8, all mice except those used as the vehicle control were sensitized via intraperitoneal injection of 20 μg OVA (Sigma Chemical Co.) and 1 mg aluminum hydroxide hydrate (Sigma Chemical Co.) in 500 μL saline. On day 21, the mice were challenged once daily for 5 days with 5% OVA for 30 min using a nebulizer (3 mL/min, NE-U17, OMRON Co. Ltd., Kyoto, Japan). During the same 5-day period, the treatment groups were also treated once daily with oral doses of dexamethasone, 25 mg/kg/day ACA, or 50 mg/kg/day ACA 1 h before the OVA challenge. After mice in the vehicle control group were sensitized with OVA, they were exposed to saline (instead of OVA) and aluminum hydroxide hydrate by nebulizer for 5 consecu\tive days. All experiments were approved by the Institutional Animal Care and Use Committee at the Korea Institute of Toxicology (Approval No. 1003-0028 & 1003-0028(1)).

### BALF analysis

One day after the final treatment, the mice were anesthetized by intraperitoneal injection of pentobarbital (60 mg/kg). The trachea was cannulated, the right lung was lavaged three times with 0.4 mL phosphate buffered saline, and the fluid was pooled. The BALF was then centrifuged at 3000 rpm for 5 min (Micro 17TR, Hanil Science Industrial Co., Ltd. Seoul, Korea), and the pellet was resuspended in 200 μL phosphate-buffered saline. Total and differential cell counts were determined by using a Coulter counter (T-540, Coulter Electronics, Hialeah, FL).

### Histopathological analysis

Lung tissues were fixed in 10% (v/v) formaldehyde solution, dehydrated in a graded ethanol series (99.9%, 90%, 80%, and 70%), and embedded in paraffin. Paraffin-embedded lung tissue was then sectioned (4 μm) longitudinally and stained with hematoxylin and eosin. Sections were also stained with PAS for semi-quantitative analysis of glycoprotein [32].

### Reverse transcription polymerase chain reaction

To evaluate proinflammatory cytokine expression, total RNA was extracted from lung tissues with the RNeasy Mini Kit (QIAGEN, Frederick, MD) according to the manufacturer's instructions. Total RNA (100 ng) was used as template for the reverse transcription reaction. Primers were synthesized for the semi-quantitative polymerase chain reaction (PCR) as follows: IL-4 forward 5′-CCAGCTAGTTGTCATCCTGC-3′, IL-4 reverse 5′-GTFATGTGGACTTGGACTCA-3′; IL-6 forward 5′-TTGCCTTCTTGGGACTGATG-3′, IL-6 reverse 5′-CAGAATTGCCATTGCACAACT-3′; IL-12α forward 5′-GCCAGGTGTCTTAGCCAGTC-3′, IL-12α reverse 5′-ATGGCCTGGAACTCTGTCTG-3′ and IL-13 forward, 5′-TCTGTGTAGCCCTGGATTCCC-3′ and reverse, 5′-CCGTGGCGAAACAGTTGCTT-3′; β-actin forward, 5′-GAAATCGTGCGTGACATC-3′ and reverse, 5′-GCTTGCTGATCCACATCT-3′. The PCR cycles consisted of denaturation at 94°C for 30 s, annealing at 58°C for 30 s and extension at 72°C for 60 s for 35 cycles. PCR products were separated by electrophoresis through a 2% agarose gel, stained with ethidium bromide, and then detected using UV light. For semi-quantitative analysis of PCR bands, the density of each band was measured with a computer imaging device and accompanying software (Bio-Rad, Hercules, CA).

### Immunohistochemical analysis

Deparaffinized tissue sections were treated with 3% hydrogen peroxide in methanol for 10 min to remove endogenous peroxidase. Antigen retrieval was carried with sodium citrate buffer (0.1 M) using the microwave method. The slides were incubated with normal serum to block nonspecific binding and then incubated overnight at 4°C with primary antibodies (diluted 1∶100–1∶200) against CD8 (Serotec, MCA55GA), CD4 (Serotec, MCA48R), CD79α (Santacruz, sc-25604), IL-13 (Santacruz, sc-73318), IL-4 (Santacruz, sc-7887), IL-5 (Santacruz, sc-9350), IL-12α (Santacruz, sc-1776), and IFN-γ (Santacruz, sc-74104). The slides were incubated for 2 h with biotinylated goat anti-rat secondary antibody (1∶500; DAKO, Carpinteria, CA) and with horseradish-peroxidase conjugated streptavidin. Signals were detected with 3,3-diaminobenzidine tetrahydrochloride substrate chromogen solution, and the cells were counterstained with Mayer's hematoxylin. To determine the number of positively stained cells, we counted cells in five randomly selected nonoverlapping fields (×200 magnification) of three separately immunostained lung sections per animal (n = 7 per group).

### Statistical analysis

Results are expressed as mean ± standard deviation (SD). Group differences were evaluated by one-way analysis of variance followed by Dunnett's multiple comparison test; *p*<0.01 was considered significant.

## References

[1] World Health Organization (May 2011) Asthma; Fact sheet N°307.

[2] Kay AB (2001) Allergy and allergic diseases. First of two parts. N Engl J Med 344: 30–37. 11136958 10.1056/NEJM200101043440106

[3] National asthma education and prevention program (2002) Expert panel report: Guidelines for the diagnosis and management of asthma update on selected topics – 2002. J Allergy Clin Immunol 110(5 Suppl): S141–219. 12542074

[4] Mosmann TR, Cherwinski H, Bond MW, Giedlin MA, Coffman RL (1986) Two types of murine helper T cell clones 1. Definition according to profiles of lymphokines activieties and secreted protein. J Immunol 136: 2348–2367. 2419430

[5] Mosmann TR, Coffiman RL (1989) Th1 and Th2 cells: differenct patterens of lymphokine secrtion lead to different functional properties. Annu Rev Immunol 7: 145–168. 2523712 10.1146/annurev.iy.07.040189.001045

[6] Rincon M, Irvin CG (2012) Role of IL-6 in asthma and other inflammatory pulmonary diseases. Int J Biol Sci 8: 1281–1290. 23136556 10.7150/ijbs.4874 PMC3491451

[7] Miteva L, Stanilova S (2008) The combined effect of interleukin (IL)-10 and IL-12 polymorphisms on induced cytokine production. Hum Immunol 69: 562–566. 18703108 10.1016/j.humimm.2008.07.008

[8] Commins SP, boorish L, Steinke JW (2010) Immunologic messenger molecules: cytokines, interferons, and chemokines. J Allergy Clin Immunol 125: S53–S72. 19932918 10.1016/j.jaci.2009.07.008

[9] Mamessier E, Magnan A (2006) Cytokines in atopic disease revisiting the Th2 dogma. 16(2): 103–113. 16581559

[10] Brightling CE, Symon FA, Birring SS, Bradding P, Pavord ID, et al (2002) Th2 cytokine expression in bronchoalveolar lavage fluid T lymphocytes and bronchial submucosa is a feature of asthma and eosinophilic bronchitis. 110(6): 899–905. 10.1067/mai.2002.129698 12464957

[11] Hershey GK (2003) IL-13 receptors and signaling pathways: an evolving web. J Allergy Clin Immunol 111: 677–690. 12704343 10.1067/mai.2003.1333

[12] Rankin JA, Picarella DE, Geba GP, Temann UA, Prasad B, et al (1996) Phenotypic and physiologic characterization of transgenic mice expressing interleukin 4 in the lung: lymphocytic and eosinophilic inflammation without airway hyperreactivity. Proc Natl Acad Sci USA 93: 7821–7825. 8755560 10.1073/pnas.93.15.7821 PMC38832

[13] Wills-Karp M, Luyimbazi J, Xu X, Schofield B, Neben TY, et al (1998) Interleukin-13: central mediator of allergic asthma. Science 282: 2258–2261. 9856949 10.1126/science.282.5397.2258

[14] Zhu Z, Homer RJ, Wang Z, Chen Q, Geba GP, et al (1999) Pulmonary expression of interleukin-13 causes inflammation, mucus hypersecretion, sub-epithelial fibrosis, physiologic abnormalities, and eotaxin production. J Clin Invest 103: 779–788. 10079098 10.1172/JCI5909 PMC408149

[15] Mattes J, Yang M, Mahalingam S, Kuehr J, Webb DC, et al (2002) Intrinsic defect in T cell production of Interleukin (IL)-13 in the absence of both IL-5 and eotaxin precludes the development of eosinophilia and airways hyperreactivity in experimental asthma. 195(11): 1433–1444. 10.1084/jem.20020009 PMC2193548 12045241

[16] Bosnjak B, Stelzmuller B, Erb KJ, Epstein MM (2011) Treatment of allergic asthma: modulation of Th2 cells and their responses. Respir Research 12: 114. 10.1186/1465-9921-12-114 PMC3179723 21867534

[17] Barnes PJ (1998) Current issues for establishing inhaled corticosteroids as the anti-inflammatory agents of choice in asthma. J Allergy Clin Immunol 101: S427–S433. 9563367 10.1016/s0091-6749(98)70154-x

[18] Kam JC, Szefler SJ, Surs W, Sher ER, Leung DY (1993) Combination IL-2 and IL-4 reduces glucocorticoid receptor-binding affinity and T cell response to glucocorticoids. J Immunol 151: 3460–3466. 8376786

[19] Warrier PK, Nambiar VPK, Ramankutty C (1993 – 1995) Indian Medicinal Plants. Orient Longman Ltd., Madras Vol. 1–5.

[20] Zheng GQ, Kenney PM, Lam LKT (1993) Potential anticarcinogenic natural products isolated from lemongrass oil and galanga root oil. J Agric Food Chem. 41(2): 153–156.

[21] Murakami A, Shigemori T, Ohigash H (2005) J Nutr Zingiberaceous and citrus constituents, 1′-Acetoxychavicol Acetate, Zerumbone, Auraptene, and Nobiletin, suppress lipopolysaccharide-induced cyclooxygenase-2 expression in RAW264.7 murine macrophages through different modes of action. 135(12 Suppl): 2987S–2982S. 10.1093/jn/135.12.2987S 16317159

[22] Ando S, Matsuda H, Morikawa T, Yoshikawa M (2005) 1′S'-1′-Acetoxychavicol acetate as a new type inhibitor of interferon-? production in lipopolysaccharide-activated mouse peritoneal macrophages. Bioorg Med Chem 13: 3289–3294. 15809164 10.1016/j.bmc.2005.02.022

[23] Matsuda H, Morikawa T, Managi H, Yoshikawa M (2003) Antiallergic principles from *Alpinia galanga*: structural requirements of phenylpropanoids for inhibition of degranulation and release of TNF-a and IL-4 in RBL-2H3 cells. Bioorg Med Chem Letters 13: 3197–3202. 10.1016/s0960-894x(03)00710-8 12951092

[24] Min HJ, Nam JW, Yu ES, Hong JH, Seo EK, et al (2009) Effect of naturally occurring hydroxychavicol acetate on the cytokine production in T helper cells. Int Immunopharmacol 9: 448–454. 19208458 10.1016/j.intimp.2009.01.008

[25] Ferreira MAR (2004) Inflammation in allergic asthma: initiating events, immunological response and risk factors. Respirology 9: 16–24. 14982597 10.1111/j.1440-1843.2003.00516.x

[26] Rothenberg ME (1998) Eosinophilia N Engl J Med. 338: 1592–1600. 10.1056/NEJM199805283382206 9603798

[27] Robinson DS, Hamid Q, Ying S, Tsicopoulos A, Barkans J, et al (1992) Predominant Th_2_-like bronchoalveolar T-lymphocyte population in atopic asthma. N Engl J Med 326: 298–304. 1530827 10.1056/NEJM199201303260504

[28] Ding J, Yang S, Xu R (1989) The inhibitory effect of hydrocortisone on interferon production by rat spleen cells. J Steroid Biochem 33(6): 1139–1141. 2615359 10.1016/0022-4731(89)90421-4

[29] Corrigan CJ, Kay AB (1991) CD4 T lymphocyte activation in acute severe asthma. Int Arch Allergy Appl Immunol 94: 270–271. 1682273 10.1159/000235380

[30] Cembrzynska-Nowak M, Szklarz E, Inglot AD, Teodorczyk-Injeyan JA (1993) Elevated release of tumor necrosis factor-alpha and interferon-gamma by bronchoalveolar leukocytes from patients with bronchial asthma. Am Rev Respir Dis 147: 291–295. 8430950 10.1164/ajrccm/147.2.291

[31] Magnan AO, Mely LG, Camilla CA, Badier MM, Montero-Julian FA, et al (2000) Assessment of the Th1/Th2 paradigm in whole blood in atopy and asthma. Increased IFN-gamma-producing CD8(+) T cells in asthma. Am J Respir Crit Care Med 161: 1790–1796. 10852746 10.1164/ajrccm.161.6.9906130

[32] Carson FL, Hladik C (2009). *Histotechnology: A Self-Instructional Text* (3 ed.). Hong Kong: American Society for Clinical Pathology Press. 137–139.

